# Polymer Waste Recycling of Injection Molding Purges with Softening for Cutting with Fresnel Solar Collector—A Real Problem Linked to Sustainability and the Circular Economy

**DOI:** 10.3390/polym16071012

**Published:** 2024-04-08

**Authors:** Ma. Guadalupe Plaza, Maria Luisa Mendoza López, José de Jesús Pérez Bueno, Joaquín Pérez Meneses, Alejandra Xochitl Maldonado Pérez

**Affiliations:** 1 Tecnológico Nacional de México, Instituto Tecnológico de Querétaro, Av. Tecnológico s/n Esq. M. Escobedo Col. Centro, Santiago de Querétaro C.P. 76000, Querétaro, Mexicojoaquin.pm@queretaro.tecnm.mx (J.P.M.); 2 Centro de Investigación y Desarrollo Tecnológico en Electroquímica, S. C., Parque Tecnológico Querétaro-Sanfandila, Pedro Escobedo C.P. 76703, Querétaro, Mexico; amaldonado@cideteq.mx

**Keywords:** recycling, purges, polymers, processing, waste

## Abstract

A plastic injection waste known as “purge” cannot be reintegrated into the recycling chain due to its shape, size, and composition. Grinding these cannot be carried out with traditional mills due to significant variations in size and shape. This work proposes a process and the design of a device that operates with solar energy to cut the purges without exceeding the degradation temperature. The size reduction allows reprocessing, revalorization, and handling. The purges are mixtures of processed polymers, so their characterization information is unavailable. Some characterizations were conducted before the design of the process and after the cut of the purges. Some of the most representative purges in a recycling company were evaluated. The flame test determines that all material mixtures retain thermoplasticity. The hardness (Shore D) presented changes in four of the purges being assessed, with results in a range of 59–71 before softening and 60–68 after softening. Young’s modulus was analyzed by the impulse excitation technique (IET), which was 2.38–3.95 GPa before softening and 1.7–4.28 after softening. The feasibility of cutting purges at their softening temperature was evaluated. This was achieved in all the purges evaluated at 250–280 °C. FTIR allowed for corroboration of no significant change in the purges after softening. The five types of purges evaluated were polypropylene-ABS, polycarbonate-ABS-polypropylene, yellow nylon 66, acetal, and black nylon 66 with fillers, and all were easily cut at their softening temperature, allowing their manipulation in subsequent process steps.

## 1. Introduction

Polymeric materials can be repeatedly recycled and reused. Gu et al. [1] studied life cycle environmental issues associated with recycling plastic waste in China. Recycled raw materials were shown to be ecologically preferable to unprocessed materials, as manufacturing products with the latter has more environmental impact than those made from reclaimed material. Recycling generally makes high-value products from waste and significantly reduces environmental impact [2,3,4,5,6]. The use of these recycled polymers in the reinforcement of construction materials is one of the alternatives that has been most evaluated in some studies [7,8,9,10,11,12,13,14,15], concluding that they present improvements in properties, such as resistance to high temperatures, mechanical durability, resistance to moisture damage, less porosity, higher resistance to cracking, higher durability, among others. 

L. Gu and Ozbakkaloglu [16] critically reviewed studies on using plastics in concrete [17,18] and found that recycled plastic fibers can improve their properties. Their review indicates that its use can contribute to a more sustainable construction industry and recommends studies focused on environmental aspects, such as the long-term behavior and ecological consequences of recycling this type of concrete after use. 

Vila-Cortavitarte et al. [19] explored the benefits of substituting polystyrene for tar in the asphalt mix. They used three types of recycled polystyrene waste in the concrete mix: general-purpose polystyrene, high-impact polystyrene, and reinforced polystyrene. This work was mainly focused on reducing the concentration of tar since its carbon emissions can pollute the environment. The mixture evaluated in this investigation was asphalt concrete. The two replacement rates of tar with polystyrene were 1% and 2% in a mix, representing about 23% and 46% of the total amount of tar. The 2% mixture was discarded due to a cohesion problem. Each sample was subjected to a series of tests to compare the modified mechanical properties. A blend with 1% polystyrene showed promising results with improved mechanical properties. The results showed that substituting polystyrene for tar reduced the environmental problem and contributed to the life cycle evaluation.

Recycling plastic waste for the construction industry is considered one of the best methods of disposing of plastic waste. Chaukura et al. [20] concentrate on some proposals for potential uses for these wastes in the manufacture of adhesives, paints, artifacts, garden furniture, and wastewater treatment products, including mosquito control. Currently, the methods for recycling plastics are grouped into primary, secondary, tertiary, and quaternary [21,22,23,24,25,26]. Since the process followed in primary and secondary recycling is the same, both are usually called “mechanical recycling”, which is frequently used [3,27]. It consists of two steps: separating the plastic from any other material and cutting it (sometimes, it is required to introduce it into an extruder to make pellets). Tertiary or chemical recycling seeks to recover the monomers or basic chemicals from the polymer. The most widely used techniques are flame, gasification, and liquefaction, although new technologies are also being developed to convert plastic waste into fuel [28,29]. Although this type of recycling is widely used, there are still many opportunities. Some works present separation analyses based on solvents or catalysts [30,31]. In general, solvent extraction separation includes removing impurities and some plastic additives, dissolution, and reprecipitation or devolatilization, which occurs when the polymers dissolve in the solvents and selectively crystallize each polymer. Selective dissolution can be used when the dissolution of the polymer of interest or all polymers except the target is feasible. The significant factor for the dissolution process is finding a selective solvent. However, several factors influence the dissolution of the polymer, such as composition, molecular weight, polymer structure, solvent composition, type, etc.

Dissolution is the most widely used technology, particularly in the automotive industry. However, it is estimated that only 20% of plastics can be recycled this way [32]. Regarding transforming polymers into hydrocarbons, Ragaert et al. [33] compiled state-of-the-art techniques, such as pyrolysis, chemolysis, hydrogen techniques, fluid catalytic cracking, and gasification. In addition, they discussed the main challenges and potential methods for this type of recycling. Finally, in quaternary recycling (known as energy recovery), as its name suggests, plastic waste is used as an energy source through incineration. Thakur et al. [34] mention that power generation by recycling plastic waste is currently one of the most eco-efficient methods, due to the massive process, and follows almost all strict emission standards and energy requirements. However, they point out that many hazardous chemicals, such as gases and dioxins, can evolve from incomplete solid waste combustion, leading to severe environmental pollution by carbon dioxide, toxic chemicals, and harmful ashes. For this reason, some incineration methods are being studied to propose those that reduce the emission of toxic substances [35,36].

Plastics have caused severe environmental pollution since they involve extracting petroleum and are thrown away. Even collecting and recycling them implies a highly energy-consuming process. Nonetheless, compared with using new raw materials, recycling is accepted as less harmful. The process of recycling plastics, even considering that there is an implicit deterioration per cycle, is an industry that follows more regulations. One of many factors in polymer environmental pollution is the extreme dependency that modern society has on them. The cost/benefit ratio for each person does not consider the collateral consequences of the items’ post-use destiny. Sustainability and the circular economy precisely consider the post-use of plastic products through purge reutilization. We are using plastics at an extreme level, considering that they degrade oppositely to glass, metal (even corroded metal can be melted again), and ceramics.

It is important to note that when any of the methods mentioned above do not recover plastic waste, it is sent to landfills. This is more than a solution; it is a severe problem due to its reduced degradability and the ample space it occupies, together with runoff and leakage of microplastics (MP) or nanoplastics (NP) [37,38]. Huang et al. [39] mentioned that landfills could have increased plastic-degrading microorganisms, causing biodegradation that, together with environmental oxidation, become drivers for plastic degradation. They show that polymers have differences in their generation rates of microplastics, with polyethylene above polypropylene (PP) or polystyrene (PS). Also, they proposed the carbonyl index to quantify the polymer degradation as a function of the disposal age. So, biodegradation through biological treatments is a widely evaluated alternative. It proposes using enzymes and organisms that can digest plastic, trap carbon, and return it to the environment as a disposable resource [4,40,41,42].

Vimala and Mathew [43] tested Bacillus subtilis for its potential to use polyethylene as its sole carbon source. They discovered that some microbial species produce surfactant compounds (biosurfactants) that enhance the degradation process. Furthermore, UV-treated polymer films facilitated their viability to feed microorganisms, which increases biodegradation.

Restrepo-Flórez et al. [44], in their study of the microbial degradation and deterioration of polyethylene, show a comprehensive summary of the microorganisms that, according to some studies, participate in the biodegradation of polyethylene. They also present their effects on the properties of polyethylene and summarize the process of its degradation. They stated that, although slow, the speed can be modulated by the intensity and physicochemical factors, such as ultraviolet light or other oxidizing agents. Therefore, the biodegradation of polymers could be a cooperative process in natural ecosystems.

Nowak et al. [45] and Mohan et al. [46] present some proposals for the biodegradation of plastics with different types of microorganisms, achieving a weight reduction of up to 2.3%. In primary, secondary, and tertiary recycling, it is important that plastic waste be as small as possible. For this, it is necessary to cut and grind it. There is a residue from the cleaning processes of plastic injection machines known as “purging” that cannot be reintegrated into the recycling chain. These are usually a mixture of polymers with some reinforcements. They have a paste consistency of size and hardness that is not possible to grind in conventional equipment due to the cost of wear and replacement of the blades, as well as the energy required for the motors. Currently, recycling companies only receive this type of material, classify it, store it temporarily, resell it, or export it. It should be noted that due to the management described, the profit is minimal, and there tend to be losses.

Derived from international agreements for the import of waste, companies have limitations for the export of purges. This generates an accumulation within the company’s facilities, with all the problems that this entails, including the lack of space, the costs of movement of the material, the prices of containers, and the severe danger due to the risk of combustion.

Some landfills are authorized to receive this type of material. However, this implies an additional environmental and economic cost to the expense already made. Also, it can only be made in restricted quantities. Likewise, various national and international technical standards describe the safety, prevention, and protection conditions for storing the material due to fire risk in the workplace, which determines having a maximum volume. Regarding quaternary recycling, there is no information on the environmental aspects that the incineration of the purge material brings. In any case, it would be necessary to characterize the material and study its emissions. Any contribution added to the recovery of polymeric waste represents an economic and environmental gain.

The case presented in this study seeks to reintegrate purges into the recycling chain through their characterization, evaluation of the conditions to reduce their size, equipment design, and softening and cutting processes that use renewable energy sources, taking care that the change processes do not degrade the material and do not significantly impact the purges that they cannot be reintegrated.

## 2. Materials and Methods

**Figure 1:**
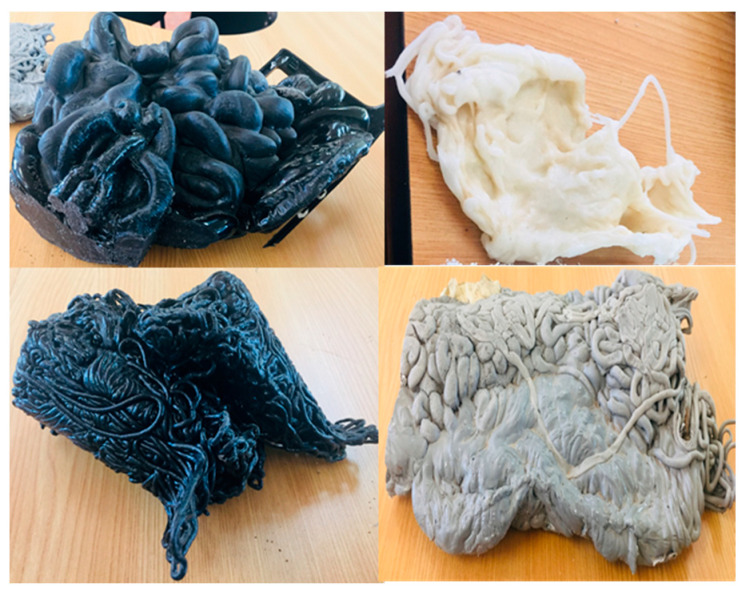
Purges of injection processes collected from industrial sources.

Purges are discarded materials in an injection process due to the cleaning carried out when there is a change in the type of part produced. The equipment is cleaned to guarantee the production of plastic objects without contamination of the previously used material, causing the residue called purge. The traditional way is to gradually introduce the new material, dragging the previous one until it is eliminated. This form of purging results in a mixture of more than one polymer, fillers, or additives with irregular sizes and shapes, some reaching up to around 30 cm per side. Figure 1 shows some purges.

This material forms with a paste consistency that solidifies in such size and hardness that it is not possible to grind it in conventional equipment. It is nearly impossible to cut purges. It is costly in terms of labor hours, security, materials, tools for use, rotary cutter, different saws (band saw, hacksaw, jigsaw), CNC router, grinder, laser cutter, waterjet cutter, plasma cutter, etc. So, exploring alternatives for industrial applications, especially renewable energies, is desirable.

### 2.1. Selection and Characterization

Five of the most frequent purges in the company Reciclajes Victoria (Queretaro, Mexico) were selected. Although all samples were from the same waste management company, they originated from different plastic processing companies. Intrinsically, purges are made of a type of polymer with or without a dye that changes from piece to piece. That condition makes the properties change from one piece to another. 

**Figure 2:**
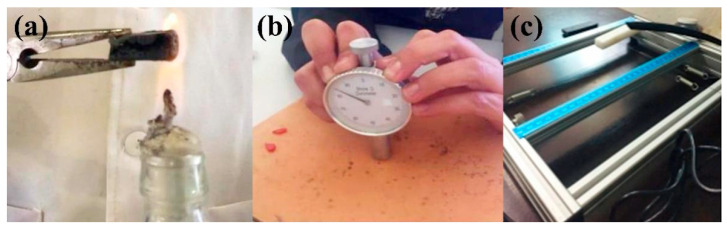
(**a**) Flame test, (**b**) hardness Shore D test, and (**c**) RFDA setup.

First, they were evaluated for how they behaved when trying to soften them and whether it was possible to cut them. Afterward, a series of characterization tests were carried out, such as flame tests, hardness, thermogravimetric analysis (TGA), Fourier transform infrared spectroscopy (FTIR), and resonant frequency damping analysis (RFDA) (used to calculate Young’s modulus). Figure 2 shows the test equipment or instruments for (a) flame, (b) hardness, and (c) RFDA.

For the flame test, there is no standard as a reference. Therefore, it was carried out by exposing a piece of a purge of approximately 1 cm^2^ to the flame of an alcohol burner, observing and taking note of all the changes in the material for about 3 min (Figure 2a).

The hardness is evaluated according to the method indicated in the ISO 868 standard [47], with a portable Shore D hardness tester TT 0.90 HD 0.25 mm. The measurement value is taken after 5 s and the result is expressed as “HSD hardness Shore D” (Figure 2b). 

The Young’s modulus was determined by the impulse excitation technique (IET) using an IMCE brand RFDA equipment. The samples for the RFDA analyses were obtained by cutting the purges using a band saw and polishing them to obtain smooth surfaces for rectangular parallelepiped pieces.

First, the bending vibration frequency was measured and Young’s modulus was calculated using the mass and dimensions of the sample according to different measurements and standard ASTM E1876-22 [48]. Next, the samples are mechanically struck by hand with a small flexible hammer. Then, the induced vibration signal is detected with a USB microphone (10 Hz–16 kHz), and later, the elastic properties are calculated using the software RFDA-MF basic v1.2.0. The calculation basis is the following relationship:

(1) $$E=0.9465\left(\frac{mF_{f}^{2}}{w}\right)\left(\frac{L^{3}}{t^{3}}\right)T$$

where, E is Young’s modulus (GPa), F_f_ is the bending frequency, m is the mass (g), w is the width (mm), L is the length (mm), t is the thickness (mm), and T is a correction factor. Figure 2c shows the experimental setup with the device bearing one sample.

Thermogravimetric analysis (TGA) was carried out to know the temperatures at which the material undergoes some degradation, so that the material does not exceed this temperature when softening. The test is carried out on a TA-Instruments (New Castle, DE, USA) SDTQ600 unit. The record produced by the analysis is a curve representing a mass variation as a function of temperature. The norm that was taken as a reference was ISO-11358 Plastics [49]. The DSC/TGA analyses were conducted using polymeric powders extracted from the purges. A hand-held electric drill was used to obtain a powder from each type of polymeric purge. The powder was treated using a Krups coffee grinder with stainless steel blades. The powder sieving was performed using a No. 400 mesh (38 μm).

Fourier transform infrared spectroscopy FTIR was carried out to determine the type of bonds and functional groups present, considering that the purges are a mixture of more than one polymer. The samples were analyzed using Bruker (Ettlingen, Germany) Tensor 37 FTIR equipment, using an ATR, in transmittance mode with 32 scans and a resolution of 1 cm^−1^. Plates of the purges (1 × 1 cm) were cut into different sections of each purge sample. They were analyzed with FTIR with coupled ATR, obtaining several spectra (1 averaged from 30 spectra per point) at different sections of the purge, and the spectra with the highest occurrence were presented.

Except for flame test, all the other tests were carried out before and after softening the purges with heating. The conditions were determined through a series of tests on samples of size around 10 × 5 × 2 cm to determine the time and temperature at which the material softens. When this happens, the pieces are cut to determine that the cut can be made easily in softening conditions. The heating was carried out in a model FE340 muffle (Felisa, Fabricantes Feligneo S.A. de C.V., Zapopan, Mexico), voltage 120 Vac, power 1500 W, frequency 60 Hz. 

### 2.2. Determination of the Processes, Design, and Manufacture of the Prototype to Soften and Cut the Purges

Using the information obtained in the characterization, the Six Sigma methodology is used to design and manufacture the prototype and the processes. Several analysis tools were used to ensure the correct design: Functional Block Diagram “DBF”, diagram “Diagram “P”, Design of experiments “DOE”, ANOVA, Hypothesis Test T, and others. The software used for data analysis were SolidWorks^®^ V2018 Modeling software and MINITAB V14 for statistical data analysis.

## 3. Results and Discussion

### 3.1. Higher Frequency Purges and Flame Test

Table 1 shows the most frequent purges in Victoria recycling, their content, description, and observations made during the flame test.

The most representative purges in Victoria recycling were polypropylene/acrylonitrile butadiene styrene (PP-ABS), polycarbonate with ABS and polypropylene (PC-ABS PP), yellow Nylon 66, white acetal (polyoxymethylene, POM), and black Nylon 66 with fillers: their main physical characteristics are described. The separation of the purges that contain mixtures of materials is not always evident. 

The flame tests were conducted to determine the general behavior of the material when heat was applied. Table 1, in its third column, shows the observations made. It is noticeable that all the polymers retain their thermoplasticity.

### 3.2. Softening and Cutting Initial Tests

The temperature at which each type of purge can be softened was determined, and the temperature was varied to evaluate the status of the materials at different times. Table 2 shows the results obtained by maintaining a constant temperature. Once the material softened, the required time was identified, it was removed from the muffle, and some observations were made about the behavior of the purge related to the cutting process.

Table 2 shows that the time for the purge containing nylon is similar to those with a higher amount, even when the quantity is less. This is because this material takes longer to soften. However, the polymers softened and cut easily in most cases.

### 3.3. Process Design and Equipment

Although the characterization tests were carried out before designing the processes to soften and cut the purges, the results are shown afterward to highlight the differences once the purges have been cut.

The overall design goal is to create processes and devices to soften and diminish the sizes of polymer purges using renewable energy sources, without exceeding the maximum temperature to avoid degradation of the polymers and keeping the design as simple as possible to facilitate its operation.

**Figure 3:**
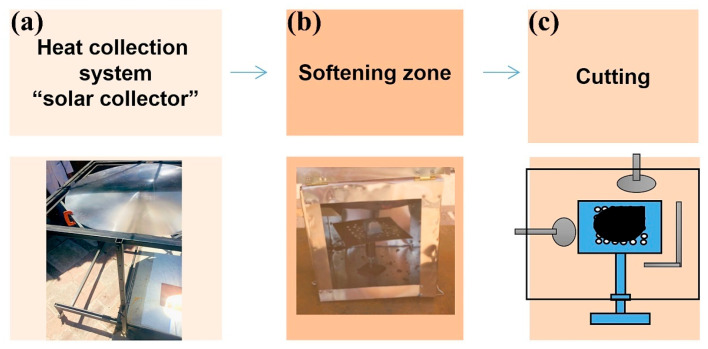
Three stages in softening plastic purges: (**a**) Concentrated solar power collector, (**b**) Heating and softening chamber, (**c**) Segmenting the pieces.

Figure 3 is a diagram, or process map. It shows the necessary processes, functions, and interactions to achieve the design objective.

Figure 3 is a process scheme showing the necessary stages, functions, and interactions to achieve the design objective. The three stages of the process identified are as follows.

(1) Heat collection system (“solar collector”). The purpose is to convert solar radiation into heat.
(2) Softening zone. The purpose is to soften the purges and maintain the temperature reached for this purpose.
(3) Cutting. Once the purges have been softened, the purpose of this last stage is to cut them off.

The design of each of these stages is explained in the following sub-sections.

### 3.4. Heat Collection System (“Solar Collector”)

#### 3.4.1. System Modeling and Construction

**Figure 4:**
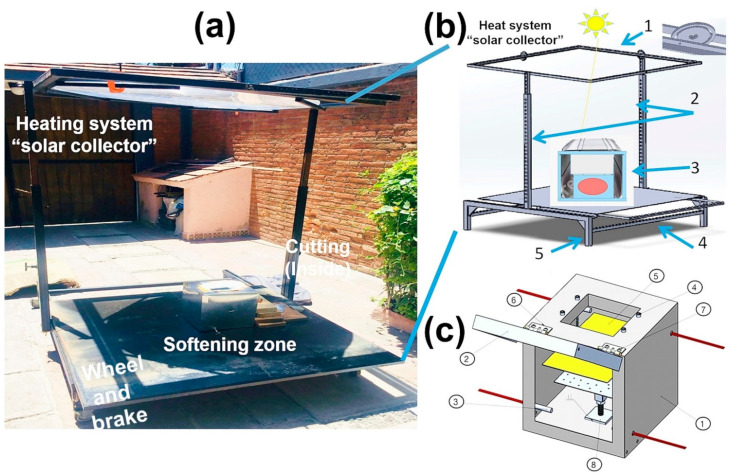
Design of the complete device. (**a**) Solar collector indicating the corresponding parts, (**b**) the collector subsystem, and (**c**) the softening zone.

The types of solar collectors were analyzed, and according to the temperature required to soften without degrading (180–280 °C), a Fresnel lens solar concentrator was used. The auxiliary components are the lens and the support. Table 3 shows the requirements and the mechanism to achieve them. Figure 4 shows the final design.

Figure 4a shows the three stages of the process (the cutting one is inside the softening zone). Figure 4b shows the attachment designed for the solar collector. The pieces that compose it are the following.

(1) Lens support consists of two frames. The inner frame supports the lens and modifies its inclination angle, referencing the position perpendicular to the height posts. The outer frame gives the lens sun-tracking capabilities.
(2) The design comprises two lateral posts, each comprising two rectangular tubes (one inside the other) that allow movement to achieve different lens heights relative to the sample. In addition, adjustment holes are spaced one inch apart for a total of twenty-six. Thus, a wide range of heights can be achieved.
(3) Plate where the softening zone was located.
(4) This plate incorporates a manual sliding system that allows it to be removed from the focus for user safety.
(5) The device supports have swivel wheels with a braking system that moves and stops the device.

#### 3.4.2. Softening Zone

The first thing that was determined for this thread was that the radiation would not be directly on the purge due to the solarization of the polymers (breaking of polymeric chains by high-intensity aces) and the difficulty in controlling the temperature. This implies the need for a means to move the heat achieved by the collector toward the softening zone. In the state of the art, the use of added thermofluids with carbon particles and air as heat transport was found [50,51,52,53,54]. This alternative was chosen due to the ease of its implementation. The study for the design of this process was carried out in stages.

Figure 4c shows the proposed heating system related to an electrical furnace. There is an internal zone in which to put the purge (cubic box). Instead of electrical resistances, they were substituted by a receptor plate for solar radiation. An internal fan was used to better distribute heat. Some other factors were addressed, such as the material and dimensions of the box, fan position, material, size, and position of the plate. Considering the required characteristics, the chosen box and plate materials were aluminum and copper, respectively. The absorbing plate could have many variants, such as using graphite, selective absorbing coatings of black nickel [55,56], black copper [57], black cobalt [58], and graphene oxide [59,60], among others. 

Figure 4c shows the proposed system for heating and cutting the purges. The components are #1 cab, #2 cab cover, #3 thermocouple, #4 m6 screws, #5 support plate, #6 hinge, #7 m5 screws, and #8 base. The absorbing plate was on the box (#5). This was fixed using four screws (#4) that adjust the distance between the plate and the plastic purge. The base for the purge (#8) rotates and has holes that allow air to flow throughout. The red lines (#3) indicate the placement of thermocouples to sense temperature in four zones. The box had a door or lid for insulation. 

Once the softening zone was manufactured, its performance was evaluated, and adjustments were made. This was carried out in two stages: (1) In conjunction with the solar collection system, (2) In evaluating the most relevant components. The focal point was circle–elliptically shaped with a diameter of about 10 cm, which means an area of about 78.54 cm^2^. The local ambient conditions were in the range of 20–27 °C and the relative humidity was about 35–45%.

Correlation studies were carried out between two factors to evaluate the functionality and interaction of the solar collection system and the softening zone. Study (A) investigated the relationship between the distance between the Fresnel lens and the receiving plate (height X), and the diameter of the radiation focus on this plate (Y_1_). Study (B) investigated the height (X) and the temperature within the softening zone or base of the samples (Y_2_). Although a relationship is expected between these variables X vs. Y_1_ and X vs. Y_2_, the objective in both studies is to find the mathematical function Y = f(x) that models this relationship and find at which value of X the highest temperature is reached, understanding that this also depends on weather conditions and exposure time (as long as it has an R factor higher than 80%). 

Table 4 shows the results obtained. The experiment conditions were the following: ambient temperature: 29 °C, relative humidity: 34.8%, pressure: 1023 hPa, solar power: 1350 W/m^2^, wind speed: 29.6 km/h, exposure time: 1 min, receiver material: copper plate, independent variable: X = height, dependent variable: Y_1_ = diameter of the focus, Y_2_ = temperature. The results were analyzed with the Minitab V14 software.

**Figure 5:**
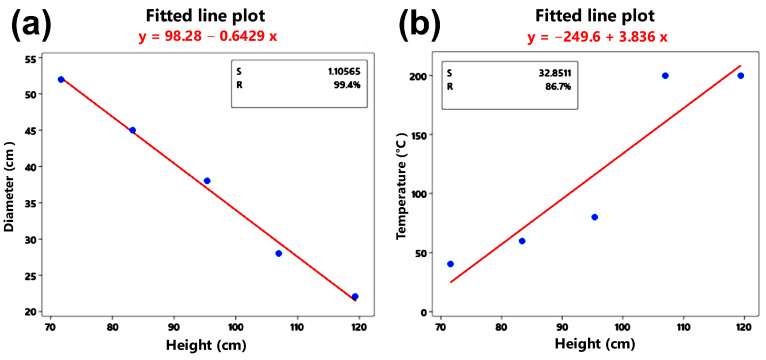
Correlation between (**a**) height and diameter and (**b**) height and temperature.

Figure 5 shows the graphic analysis of the height–temperature correlation and height analysis vs. diameter (X vs. Y_1_).

Figure 5a shows that the greater the height X, the diameter of the radiation focus decreases linearly. Therefore, the mathematical model is valid since the relationship has a factor higher than 80% with an adjustment value of R = 99.2% and a variance of 1.10. Diameter = 98.28 cm and height [cm] = 0.643 cm. Table 5 shows the ANOVA analysis of variance.

Considering the value of *p*, which is less than the value of the statistical error α = 0.05, *p* = 0.022 < α = 0.05, it is concluded that the temperature Y_2_ depends directly on the height X. 

Figure 5b graphically shows the correlation between X and Y_2_. It shows that the higher the height X, the more the temperature increases linearly. Therefore, with an adjustment value of R = 82.3% and a variance of 1.10, the mathematical model is valid since the relationship has a factor greater than 80%. Temperature = 249.6 °C and height [cm] = 3.836 cm.

Using the prediction equation, the temperature-maximizing height is about 120 cm. According to the holes that regulate the height, an elevation of about 122 cm was used in tests.

#### 3.4.3. Evaluating the Most Relevant Components of the Softening Zone 

Two relevant elements are observed for the scope and temperature control: (1) The fan use. (2) The material of the radiation receptor plate.

Through a design of experiments, these factors are evaluated, determining the impact on the temperature reached in four points of the box and finding the condition that maximizes the temperature in the position of the base or central position (that shortens the time to soften the purge). Table 6 shows the DDE approach.

Table 6 shows the approach introduced in the Minitab V14 software, DDE: two factors (fan and receptor material), two replicates, without central points, and all in a single block, giving ten experiments. The experimental conditions were as follows. Ambient temperature: 18 °C, relative humidity: 63.6%, pressure: 1025 hPa, solar power: 952 W/m^2^, wind speed: 0 km/h, exposure time: 2 min, X_1_ = fan, evaluating using it or not, X_2_ = material of the absorbing plate, evaluating a copper or steel sheet. The dependent variables were as follows. Y_1_ = Upper right position temperature, Y_2_ = Central position temperature, Y_3_ = Upper left position temperature, Y_4_ = Lower left position temperature, Y_5_ = Lower right position temperature. 

**Figure 6:**
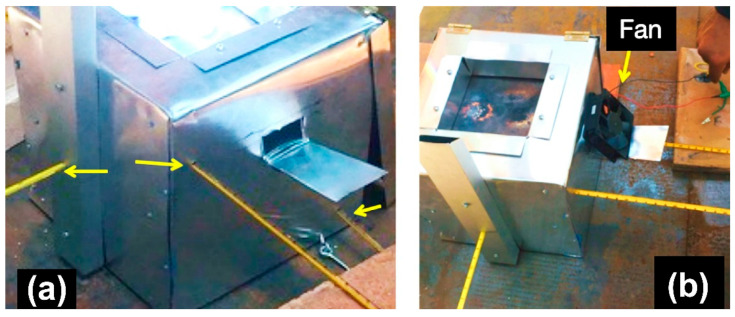
(**a**) Location of the thermocouples (indicated with yellow arrows). (**b**) Location of fan.

Figure 6a shows the position of the thermocouples on one of the sides and the center (the other side has the same position), and Figure 6b shows the fan.

Table 7 shows, in the “Test run” column, the order of the experiments (random order), while the “Fan” and “Receiving sheet” columns show the conditions under which the corresponding experiment is carried out. The Y_1_–Y_5_ columns correspond to the temperature °C obtained under each evaluated condition. Table 7 shows the analysis of variance (ANOVA) for Y_1_ = Upper right position temperature.

Table 8 shows the ANOVA analysis of variance. Considering the value of the *p* statistic, Fan *p* = 0.022, receiver material *p* = 0.003, they are less than the statistical error α = 0.05, with which it is concluded that both variables influence linearly the temperature obtained in position Y_1._ The interaction of these *p* = 0.075 is above the error; therefore, there is no interaction. The same ANOVA analysis was performed for each thermocouple position (Y_2_–Y_5_) and the results are summarized in Table 9.

The first column, Y, indicates the thermocouple position. The second to fourth indicate if the X factor, or the interaction of these, influences the results of the Y. The fifth indicates which of the X has the greatest influence. The last two columns indicate the combination with which the highest temperature is obtained.

In conclusion to this analysis, we can say that in the central and upper zones, the temperature is affected by the type of receiving material and the use, or not, of the fan; in the lower part, it only affects the use, or not, of the fan. To evaluate the configuration that maximizes the temperature, only position Y_2_ is considered; that is, the central area where the sample is placed. The result is “Copper receptor material” and “Without fan”.

### 3.5. Hardness Testing

Thirty hardness measurements were made in different areas of the purge to determine material homogeneity before and after material softening. The t-student statistic test was applied with a 95% confidence level and a hypothesis approach. Null hypothesis H0: µ1 − µ2 = 0, alternative hypothesis H1: µ1 − µ2 ≠ 0.

The hypothesis test is carried out with the test statistic of the *p* value to evaluate if there is a statistical difference in the mean hardness values before and after the heat treatment of the sample. An error value of 0.05 is considered to accept or reject the null hypothesis, assuming normal data for this number of measurements. On the other hand, the Levene statistical test is applied to evaluate the impact on the variation in the results obtained in both conditions for each of the purges. Table 10 shows the results obtained and the conclusion of the statistical analysis.

**Figure 7:**
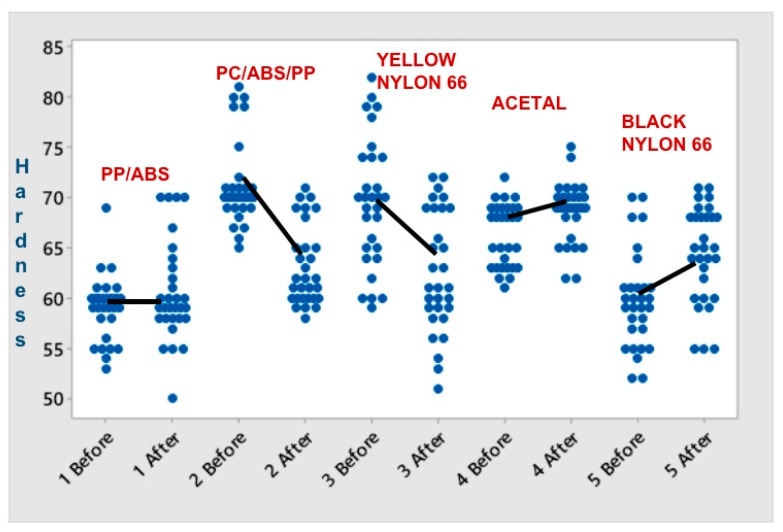
Distribution and averages of hardness values before and after softening.

In the conclusions of the averages analysis, we can see no changes in PP-ABS, PC-ABS-PP, and Nylon 66 yellow. Their hardness decreases after softening, while Acetal and Nylon 66/black with fillers hardness increases. Variation does not change in any of the samples. Figure 7 shows how the data are distributed before and after softening each purge type.

Figure 7 shows that the data have an equal distribution before and after the softening of the material. There was a slight difference between the means in all purges.

### 3.6. Young’s Module

Table 11 shows the results of the measurements made before and after softening the material.

**Figure 8:**
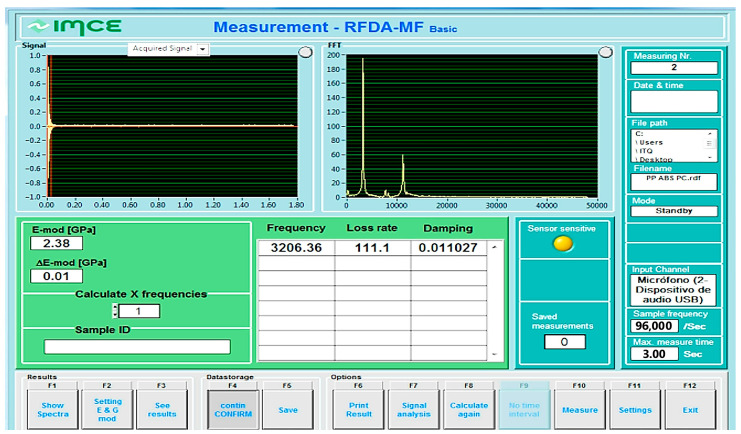
RFDA test set results screen.

Table 11 shows the average of three data values taken for each purge before and after softening and cutting. The last column indicates with an arrow if the average increased or decreased, and by what percentage. For example, in PP-ABS, Young’s modulus decreases by 18% after smoothing and cutting. In general, considering that the purges are a recycling material, the variation is slight in all the samples. Figure 8 shows how the test device reports results.

**Figure 9:**
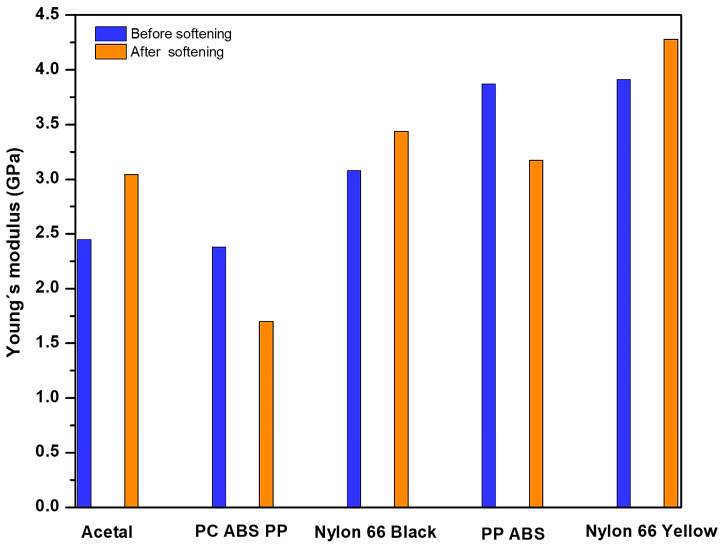
Comparison of Young’s modulus average before and after softening.

Figure 8 shows the graph of the noise signal in the upper part. The lower left part shows Young’s modulus. The rest of the information shows the data the software uses for the calculation. Figure 9 shows the results in a bar graph.

Figure 9 shows the results in a bar graph, showing the difference between the average values before and after softening and cutting each purge.

### 3.7. DSC and TGA Analyses

The glass transition temperature (T_g_) for ABS typically ranges from 105 to 125 °C, depending on the proportions of the three components and the molecular weights of the polymeric chains. Variations in the T_g_ depend on specific formulation and processing conditions in any polymer. Still, they are even more frequent in industrial polymer purges that could have plasticizers, colorants, or broader molecular weights.

The T_g_ of PC is in the range of 145–150 °C. Nylon 66, PP, and Acetal (polyoxymethylene) are semi-crystalline polymers in which the T_g_ is the transition from a glassy to a rubbery state in the range 45–50 °C, −10 to −5 °C, and −50 to −80 °C, respectively.

**Figure 10:**
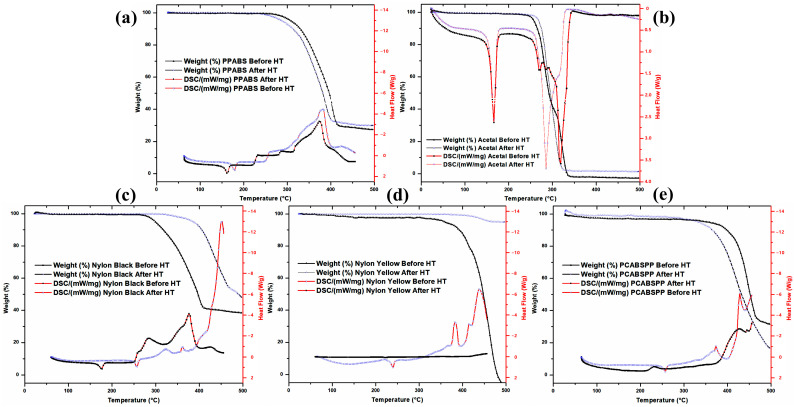
DSC and TGA analyses for the five polymers studied, showing their graphs before and after the heat treatments by indirect sunlight heating (**a**) PP ABS, (**b**) Acetal, (**c**) Nylon 66 black, (**d**) Nylon 66 yellow, and (**e**) PC ABS PP.

DSC/TGA calorimetry analyses were performed to determine the maximum temperatures for heating. As long as neither the DSC graphs show irreversible events, nor the TGA graphs show weight loss, heating can be conducted while maintaining the polymer’s integrity and original polymer characteristics. For example, in Figure 10a PP ABS, Figure 10b Acetal, Figure 10c Nylon 66 black, Figure 10d Nylon 66 yellow, and Figure 10e PC ABS PP, the proposed maximum temperature could be about 245, 240, 250, 330, and 270 °C, respectively.

### 3.8. FT-IR Analyses

**Figure 11:**
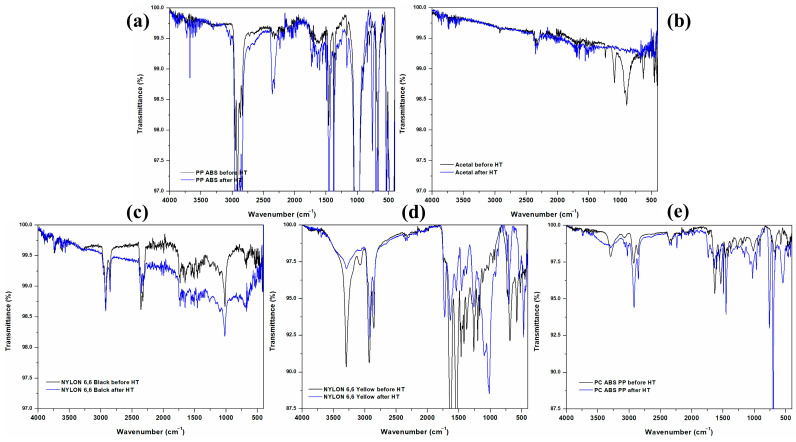
FT-IR spectra for the five polymers studied, showing their vibrational bands before and after the heat treatments by indirect sunlight heating (**a**) PP ABS, (**b**) Acetal, (**c**) Nylon 66 black, (**d**) Nylon 66 yellow, and (**e**) PC ABS PP.

The five polymers have functional groups with characteristic vibrational bands in their FT-IR spectra (Figure 11). Some of the representative vibrational bands are outlined below.

Polypropylene (PP) has C-H stretching and bending vibrations and C-C stretching in the ranges of 2800–3000, 1350–1470, and 960–970 cm^−1^, respectively. Also, PP has bands related to its isotactic configuration, such as isotactic CH_3_ bending vibrations in the region of 875–885 cm^−1^ (Figure 11a,e).

The terpolymer ABS (Acrylonitrile–Butadiene–Styrene, 3–7: 1–6: 8–12) has a C-H backbone with stretching and bending vibrations in the 2900–3000 and 1300–1400 cm^−1^ (Figure 11a,e) [61,62,63] regions. Acrylonitrile cyano group’s C≡N stretching vibrations are around 2240–2260 cm^−1^. Butadiene C=C stretching and CH_2_ deformation vibrations are in the regions of 1660–1600 and 1450–1375 cm^−1^, respectively. Styrene vibrations of the aromatic ring deformations are in the range of 700–750 cm^−1^.

Acetal (polyoxymethylene) has C-H, C=O, C-O, and O-CH_2_-O stretching vibrations in the regions of 2900–3000, 1720–1740, 1050–1150, and 1150–1250 cm^−1^ associated with CH_2_ groups, acetal groups, polymer backbone, and ether linkage, respectively (Figure 11b).

Nylon 66 (polyhexamethylene adipamide) has N-H bending vibrations and N-H, C=O, and C-N stretching vibrations. There are bands of Amide A, Amide B, Amide I, Amide II, and Amide III in the 3200–3500, 1550–1650, 1620–1660, 1500–1550, and 1200–1300 cm^−1^ ranges, respectively (Figure 11c,d).

Polycarbonate (PC) has the C-H stretching and carbonate ester stretching and bending vibrations in the ranges of 2900–3100, 1740–1760, and 700–800 cm^−1^, respectively (Figure 11e).

The solar-assisted thermal softening treatment performed in the polymers decreased the intensity of some vibrational bands. The proposed treatment is intended to affect the functional groups and the polymeric chain lengths as little as possible. That was the reason for conducting the DSC/TGA calorimetry analyses shown above to determine the limit temperatures for heating. The heating can be carried out and repeated as long as the original polymer characteristics can be maintained barely unchanged. 

## 4. Conclusions

Plastic injection purges have many sizes and irregular shapes that require reductive changes to be recycled and reintegrated into the polymer supply chains. For this, it is important to know their characteristics, properties, behavior, and grouping by polymer type. Frequency injection purges are blends of processed polymers that are difficult to recycle. This work proposes a process and a prototype device for softening and cutting the injection purges using solar heating. In this study, the starting tested samples were about 30 × 25 × 20 cm in size, and the final size was set to about 10 × 5 × 2 cm.

One of the problems with purges is the combination of materials, which does not allow incorporation into the manufacturing process. Other issues are the complex shapes and properties of the purges, which reduce the possibility of re-insertion into the process. The softening of the material and the feasibility of cutting the pieces with minimal impact on the polymer structure do allow the circularity of the material. There is less waste of materials with reintegration into production, which is the basis of the circular economy.

The thermal properties were evaluated using the flame test, TGA, and DSC techniques. The conclusive results are that none of the purges leak during heating, the five types of studied polymers can stretch, and their softening temperatures are between 250 °C and 280 °C. Once all the evaluated purges softened, they were cut relatively easily. Visually, no smoke was observed when reaching a soft condition. Using this information, a process and a device that operates with solar energy were designed to cut the purges without exceeding the maximum temperature, which varied depending on the type and quantity of polymers. Finally, the mechanical properties of hardness and Young’s modulus were evaluated, and the FTIR tests were carried out before and after softening and cutting. Therefore, any change in the polymers could be related to reticulation or chain breakage, which are not associated with reaching stability but with deterioration. Knowing the calorimetry graphs, the maximum limit for the temperature was proposed for each polymer where the TGA graph deviates from a horizontal line. PP ABS, Acetal, Nylon 66 black, Nylon 66 yellow, and PC ABS PP could have, as proposed maximum temperatures for softening and cutting into smaller pieces, about 245, 240, 250, 330, and 270 °C, respectively.

Comparisons of the mechanical properties of the purges before and after treatments showed slight affectation on most of them. Generally, the main functional groups did not change. Further improvements are advisable in both the process and the device. Still, this work shows potential viability for solar-assisted heating for softening and cutting injection purges that constitute a fast-increasing residue of the plastics industry, making them a byproduct to be reprocessed.

This work differs from other research or industry solutions because it aims to soften the pieces to obtain smaller, specific-size elements rather than use them as fillers, composites, or recycling by reprocessing to obtain products such as bottles or fabrics. Considering that the main concern is keeping polymer integrity but softening it, this process can be extended to most organic polymers. Nonetheless, the size of the pieces is important for the system characteristics. Previous experiences advise specializing such a system for a single type of polymer, but if this is not possible, a technical procedure could be elaborated to change the temperature and keep the process clean when changing the materials to be processed.

In the proposed system, each part has a different expected lifespan. This largely depends on factors such as materials, process operation ranges, maintenance, surrounding climatic conditions, and the observation of safety rules during process operation, among others.

## Figures and Tables

**Table 1:** Some representative industrial purges from polymer injection.

Polymers	Physical Description	Observations	Image
PP-ABS	Gray with many marked lines	The material quickly deforms, the flame is yellow and blue, bubbles, and becomes rubbery but does not drip, and carbonizes in its walls. As a result, it can be stretched and makes almost no smoke.	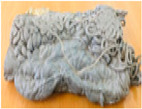
PC-ABS-PP	Two shades of black (one glossy and one opaque)	Quickly begins to burn and deform. The flame is yellow, it causes fumes of black smoke, and has a marked odor. It becomes rubbery, but drips cannot be stretched and carbonized.	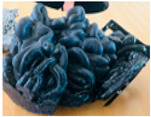
Nylon 66 yellow	Yellow with many marked lines	It takes time to begin to burn and change its form. Then it bubbles, causes a small amount of fumes, the flame is yellow and blue, becomes rubbery but does not drip, can be stretched, and gets carbonized at the end.	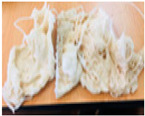
Acetal (polyoxymethylene, POM)	White with many grains	Quickly begins to burn and deform. The flame is yellow and blue, and it causes a small amount of fumes. It has a marked odor. It bubbles a lot but does not drip, can be stretched, becomes rubbery, and gets carbonized at the end.	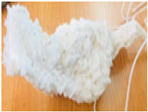
Nylon 66/black with fillers	Spaghetti-shaped black	It takes time to begin to burn and change its form. It bubbles but does not drip, making almost no smoke. The flame is yellow and blue, hardly stretched, and nearly no carbonization at the end.	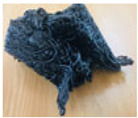

**Table 2:** Softening and cutting tests.

Polymers	Weight (g)	Temperature (°C)	Time (min)	Observations
PP-ABS	16.7	250	20	It can be easily cut, and it can be stretched, but it tends to become rubbery.
PC-ABS-PP	52.99	250	20	It can be easily cut and stretched.
Nylon 66 yellow	6.64	250	20	It cools down very fast and hardens. It can be stretched. Changes to a dark color. It is relatively difficult to cut and has a sandy consistency.
Acetal (polyoxymethylene, POM)	29.88	300	45	It cools down very fast and hardens. It can be stretched. Changes to a dark color. Difficult to cut.
Nylon 66/black with fillers	19.63	300	50	It cools down very fast and hardens. It can be stretched but has a dusty consistency.

**Table 3:** Requirements and the mechanism to achieve them.

Requirements	Answer
Increase or decrease the diameter of the focus	Height adjustment mechanism
Adjusted to the position of the sun	Angle adjustment mechanism
A softening zone	Sample tray
Easy to move	Drive wheels and brake
No risk of the lens falling off	Padded retainer
Samples can be inserted and removed without risk of burns	Handle—endless screw system or rails

**Table 4:** The correlation results.

Height (cm)	Diameter (cm)	Temp. (°C)
71.5	52	40
83.3	45	60
95.3	38	80
107	28	200
119.5	22	200

**Table 5:** ANOVA for the Height–Temperature calculus and the Height vs. Diameter.

Source	GL	MC	F	P	GL	SC	MC	F	P
Regression	1	21,082	19.54	0.022	1	592.333	592.333	484.54	0
Height [cm]	1	21,082	19.54	0.022					
Error	3	1079			3	3.667	1.222		
Total	4				4	596			
Source	GL	MC	F	P	3	3.667	1.222		

**Table 6:** Approach to the design of experiments.

Factors	2	Base design	2, 4	Factor	Level +	Level −
Test runs	8	Replica	2	Fan	With	Without
Blocks	1	Center points (total)	0	Absorbing material	Copper	Steel sheet

**Table 7:** Shows the test plan with the results obtained.

Test Run	Fan	Receiving Sheet	Y_1_	Y_2_	Y_3_	Y_4_	Y_5_
1	No	galvanized	74	98	79	84	90
2	Yes	galvanized	74	78	72	72	82
3	No	copper	110	130	104	90	106
4	Yes	galvanized	76	76	76	80	90
5	Yes	copper	92	80	80	72	80
6	Yes	copper	90	82	80	74	80
7	No	copper	120	130	110	88	100
8	No	galvanized	86	104	86	94	100

**Table 8:** ANOVA for Y_1_.

Source	DF	Adj SS	Adj MS	F-Value	*p*-Value
Model	3	1901.5	633.83	20.12	0.007
Linear	2	1721	860.5	27.32	0.005
Fan	1	420.5	420.5	13.35	0.022
Receiving sheet	1	1300.5	1300.5	41.29	0.003
2-Way Interactions	1	180.5	180.5	5.73	0.075
Fan * receiving sheet	1	180.5	180.5	5.73	0.075
Error	4	126	31.5		
Total	7	2027.5			

* Interaction between Fan and Receiving sheet.

**Table 9:** Summary of temperature results in the thermocouples’ (Y’s) positions.

Y	Fan X_1_	Material X_2_	Interaction	X with More Influence	Best Combination	
					X_1_	X_2_
Y_1_= Upper right position temperature	Yes	Yes	No	Material	Without	Copper
Y_2_ = Central position temperature	Yes	Yes	Yes	Fan	Without	Copper
Y_3_ = Upper left position temperature	Yes	Yes	Yes	Fan	Without	Copper
Y_4_ = Lower left position temperature	Yes	No	Yes	Fan	Without	Same
Y_5_ = Lower right position temperature	Yes	No	No	Fan	Without	Same

**Table 10:** Hardness analyses (Shore D) of five polymer purge types with thirty measured samples.

Polymers	Average Hardness before Heating	Average Hardness after Heating–Cooling	Conclusions of the Average Analyses	Conclusions of the Variance Analyses
PP-ABS	59.03	60.6	*p*-value = 0.149 No change	*p*-value = 0.115 No change
PC-ABS-PP	71.33	63.23	*p*-value = 0.00 Different after the heat decreases	*p*-value = 0.591 No change
Nylon 66 yellow	69.53	62.63	*p*-value = 0.001 Different after the heat decreases	*p*-value = 0.364 No change
Acetal	66.37	68.67	*p*-value = 0.005 Different after the heat increases	*p*-value = 0.218 No change
Nylon 66/black with fillers	59.8	64.43	*p*-value = 0.00 Different after the heat increases	*p*-value = 0.701 No change

**Table 11:** Young’s modulus (GPa).

Polymers	YM before	YM after	Comparative
PP-ABS	3.88 (±0.02)	3.17 (±0.01)	↓ 18%
PC-ABS-PP	2.38 (±0.001)	1.70 (±0.001)	↓ 28%
Nylon 66 yellow	3.94 (±0.01)	4.28 (±0.01)	↑ 9%
Acetal (polyoxymethylene, POM)	2.45 (±0.001)	3.04 (±0.01)	↑ 24%
Nylon 66/black with fillers	3.08 (±0.001)	3.45 (±0.005)	↑ 12%

Arrow down: YM decreases, Arrow up: YM increases.

## Data Availability

Data are contained within the article.
